# Activation of Nicotinic Acetylcholine α7 Receptor Attenuates Progression of Monocrotaline-Induced Pulmonary Hypertension in Rats by Downregulating the NLRP3 Inflammasome

**DOI:** 10.3389/fphar.2019.00128

**Published:** 2019-02-26

**Authors:** Yan Deng, Sheng-Lan Guo, Bin Wei, Xing-Cui Gao, Ying-Chuan Zhou, Jia-Quan Li

**Affiliations:** ^1^Department of Ultrasound, The Cardiovascular Disease Institute, The First Affiliated Hospital to Guangxi Medical University, Nanning, China; ^2^Department of Cardiology, The First Affiliated Hospital to Guangxi Medical University, Nanning, China; ^3^Department of Pathology, The First Affiliated Hospital to Guangxi Medical University, Nanning, China; ^4^The Experimental Center of Guangxi Medical University, Nanning, China

**Keywords:** pulmonary hypertension, α7nACh nicotinic acetylcholine receptor, NLRP3 inflammasome, pulmonary vascular remodeling, inflammation

## Abstract

**Background:** Inflammation and altered immunity contribute to the development of pulmonary arterial hypertension (PH). The alpha 7 nicotinic acetylcholine receptor (α7nAChR) possesses anti-inflammatory activities. The current study was performed to investigate the effects of a selective α7nAChR agonist, PNU-282987, on controlling a monocrotaline (MCT)-induced rat model of PH and explored the underlying mechanisms.

**Methods:** Sprague-Dawley rats were injected with MCT and treated with PNU-282987 at the prevention (starting 1 week before MCT) and treatment (starting 2 weeks after MCT) settings. Four weeks after MCT injection, hemodynamic changes, right ventricular structure, and lung morphological features were assessed. Enzyme-linked immunosorbent assay, Western blot and *q*RT-PCR were performed to assess levels of inflammatory cytokines and NLRP3 (Nod-like receptor family pyrin domain-containing 3) inflammasome pathway in the rat lung tissues. In addition, the lung macrophage line NR8383 was used to confirm the *in vivo* data.

**Results:** Monocrotaline injection produced PH in rats and downregulated α7nAChR mRNA and protein expression in rat lung tissues compared to sham controls. Pharmacological activation of α7nAChR by PNU-282987 therapy improved the rat survival rate, attenuated the development of PH as assessed by remodeling of pulmonary arterioles, reduced the right ventricular (RV) systolic pressure, and ameliorated the hypertrophy and fibrosis of the RV in rats with MCT-induced PH. The expression of TNF-α, IL-6, IL-1β, and IL-18 were downregulated in rat lung tissues, which implied that PNU-282987 therapy may help regulate inflammation. These protective effects involved the inhibition of the NLRP3 inflammasome. *In vitro* assays of cultured rat lung macrophages confirmed that the anti-inflammation effect of PNU-282987 therapy may contribute to the disturbance of NLRP3 inflammasome activation.

**Conclusion:** Targeting α7nAChR with PNU-282987 could effectively prevent and treat PH with benefits for preventing ongoing inflammation in the lungs of rats with MCT-induced PH by inhibiting NLRP3 inflammasome activation.

## Introduction

Pulmonary hypertension (PH) is characterized by progressive pulmonary vascular remodeling, microvascular loss, and elevated pulmonary vascular resistance leading to increased right-sided heart failure and high rates of morbidity and mortality (No authors, 2015; Potus et al., 2015; Mendes-Ferreira et al., 2016). PH was once thought to be caused by increased vasoconstrictor tone; however, the use of vasodilators is insufficient to overcome the inevitable disease progression in the majority of patients (Rabinovitch, 2012). Excessive pulmonary vascular inflammation, along with the recruitment and infiltration of circulating inflammatory cells, are considered to play an important role in the pathogenesis of PH (Rabinovitch, 2012; Rabinovitch et al., 2014; Gurtu and Michelakis, 2015).

Lung macrophages are the key pathogenic drivers involved in orchestrating both the initiation and resolution of pulmonary inflammation (Huang W. C. et al., 2016; Florentin et al., 2018). Macrophages are generated from circulating monocytes and then differentiate into macrophages, which produce inflammatory cytokines, thereby promoting pulmonary artery smooth muscle cells (PASMC) proliferation via growth factor signaling (Gurtu and Michelakis, 2015; Florentin et al., 2018). The role of a macrophage is dependent on its microenvironment, with which it can interact through its large repertoire of surface receptors (Zhou et al., 2014; Layhadi and Fountain, 2017; Rojas et al., 2018). In this regard, identifying and targeting inflammatory “upstream” receptors could represent novel therapeutic strategies.

A crosstalk has been identified between the immune system and the vagus nerve to modulate systemic and local immune responses in different systems, including respiratory and cardiovascular (Marrero et al., 2011; Liu and Su, 2012). Alpha7 nicotinic acetylcholine receptor (α7nAChR), firstly identified in the autonomous nervous system, is a ligand-gated ion channel exerted as a regulator in cognitive processes through the modulation of specific neurotransmitters (Marrero et al., 2011; Liu and Su, 2012; Lu et al., 2014). More recently, resident macrophages in different tissues proved to highly express α7nAChR, and the activation of this receptor inhibits the production of inflammatory cytokines, thereby attenuating the local inflammatory response (Yu et al., 2013; Foucault-Fruchard et al., 2018; Meroni et al., 2018). Previous studies demonstrated that the α7nAChR activation mediated bowel, neural, and cardiovascular protection (Yu et al., 2013; Foucault-Fruchard et al., 2018; Meroni et al., 2018). Consistent with these data, the administration of α7nAChR agonist has been shown to effectively prevent acute lung injury via inflammation suppression (Ge et al., 2017; Pinheiro et al., 2017).

Nod-like receptor family pyrin domain-containing 3 (NLRP3) inflammasome, comprising the NLRP3, the apoptosis speck-like protein containing a caspase-recruitment domain (ASC), and pro-caspase-1, mediate cytokine and inflammatory responses in PH (Tang et al., 2015; Yin et al., 2017). The NLRP3 inflammasome is predominantly expressed in macrophages and functions as a multiple cytoplasmic sensor molecule (Mangan et al., 2018). Recent studies indicated that α7nAChR inhibition-triggered inflammation cascades may occur through the suppression of the NLRP3 inflammasome in microglia (Lu et al., 2014). Hence, we speculated that the role of α7nAChR in regulating the NLRP3 inflammasome may be involved in the development of PH.

Monocrotaline (MCT),11-member macrocyclic pyrrolizidine alkaloid derived from the seeds of the crotalaria spectabilis plant, induce PH in rats similar to human PH associated with acute lung injury (Gomez-Arroyo et al., 2012; Hill et al., 2017). This animal model is useful in studying the pathogenesis and the pre-clinical assessment of novel therapeutics for PH (Huang Z. et al., 2016; Deng et al., 2017; Hill et al., 2017; Zhao et al., 2017). A previous study revealed that activation of α7nAChR using a small molecule agonist was effective in inhibiting inflammation factors released from the macrophage lineage in a acute lung injury model (Ge et al., 2017). In this study, we explore whether the selective α7nAChR agonist, PNU-282987, inhibiting the progression of PH in a rat MCT model and the underlying mechanisms.

## Materials and Methods

### Animals

This study was performed according to the Guide for the Care and Use of Laboratory Animals (NIH Publication No. 85-23, revised 1996), and an animal protocol was approved by the Institutional Animal Care and Use Committee (IACUC) of Guangxi Medical University (Nanning, China) with the approval certificate #0062353-SCXK (SH) 2007-0005. Male Sprague-Dawley rats weighing at 260–300 g were obtained from the Experimental Laboratory Animal Center of Guangxi Medical University and housed in a specific pathogen-free “barrier” facility with standard housing conditions (room temperature: 21–22°C) with 60–65% humidity and free access to food and water *ad libitum*x.

### Animal Experiments

PNU-282987 was purchased from Sigma Chemical, Co. (St. Louis, MO, United States) and dissolved in 0.9% saline before use. For our animal experiments, 60 rats were randomly divided into four groups to receive a single subcutaneous injection of 60 mg/kg monocrotaline (Sigma Chemical, Co.) or normal saline as a vehicle control: (1) Sham group, saline + vehicle (*n* = 15); (2) MCT group, MCT + vehicle (*n* = 15); (3) Prevention group, MCT + PNU-282987 (4.8 mg/kg) (*n* = 15), in which MCT rats received continuous daily intraperitoneal injection of PNU-282987 1 week before MCT administration; and (4) Treatment group, MCT + PNU-282987 (4.8 mg/kg) (*n* = 15), in which the MCT rats received a continuous daily intraperitoneal injection of PNU-282987 2 weeks after MCT administration. The dose selection of PNU-282987 in this study was based on a previous animal study (Ge et al., 2017). The vehicle and MCT were administrated only once, while the preventive or treatment doses of PNU-282987 were given daily until the fourth week after MCT administration.

### Echocardiography and Hemodynamic Measurement

In the MCT rat model of PH, rat pulmonary vascular and hemodynamic obvious alterations and the RV remodeling occurred in 28 days after MCT injection (Gomez-Arroyo et al., 2012; Deng et al., 2017). In this study, we performed echocardiography and hemodynamic evaluations at this period of time. The rats were anesthetized through the intraperitoneal injection of ketamine (75 mg/kg), and the rat body temperature was kept at 37°C using a heating pad. Thereafter, a transthoracic echocardiography was performed using a HP 7500 system with a 12S transducer (Philips, Hewlett-Packard, Co., Andover, MA, United States) to measure the RV end diastolic dimension (RVEDD) and tricuspid annular plane systolic excursion (TAPSE) according to a previous study (Deng et al., 2017). The measurement recorded for 10 consecutive heartbeats and was normalized for beat-to-beat variations.

After that, we performed a direct RV puncture with a 21-gauge needle attached to a pressure transducer (ALCB10 Heart Function Analysis System; Shanghai Alcott Biotech, Co. Ltd., China) to measure the RV systolic pressure (RVSP) for at least 6 s with a stable pressure waveform. The pulmonary artery systolic pressure was equivalent to the RVSP when excluding the RV outflow tract and pulmonary valve stenosis under echocardiography (Rocchetti et al., 2014).

### Tissues and Histological Examination

After hemodynamic procedures, the rats were euthanized for resection of the heart and lung tissues, which were then weighed to assess the dry weight of the RV free wall, the LV plus septum (LV + S). The ratio of the RV free wall to the free LV wall and the ventricular septum (RV/LV+S) and the ratio of the dry weight of RV to the body weight (RV/BW) were calculated. Partial RV and inflated left lung tissues of all rats were submerged in ice-cold saline, subsequently fixed in 4% paraformaldehyde through perfusion for 24 h, and embedded in paraffin for sectioning, hematoxylin-eosin (H&E) staining, and histopathological examination. Meanwhile, the left lung and RV tissues were snap-frozen in liquid nitrogen and stored at -80°C for future enzyme-linked immunosorbent assay (ELISA), *q*RT-PCR, and Western blot analyses.

Specifically, lung tissue sections with 5-μm thickness were stained with H&E. RV myocardial tissue sections with 5-μm thickness were stained with Masson trichome staining according to the kit instructions (SenBeiJia Biological Technology, Co., Ltd., Nanjing, China). Tissues were reviewed, and the images were captured under an Olympus BX51 microscope equipped with analysis software (Olympus, Tokyo, Japan) for the median vessel wall thickness (for vessel occlusion), which was calculated by the ratio of the intra-acinar pulmonary vessels of (the outer vessel area – the luminal area)/(the outer vessel area) in small pulmonary arterioles with an outer diameter of 50–150 μm according to a previous study (Rocchetti et al., 2014). For each animal, over 20 randomly selected vessels that were closed to a round or oval shape were measured and averaged by two investigators in a blinded fashion. Moreover, the interstitial collagen deposition in the RV free wall was also assessed as the percentage of fibrous tissue area in the visual field in Masson-trichrome stained lung tissue sections. The photo images of these lung tissue sections were analyzed using the Image-Pro Plus 6.0 (Media Cybernetics, Silver Spring, MD, United States) by two pathology experts for 10 randomly selected microscopic fields (magnification, x400) for each section.

### Immunohistochemistry and Immunofluorescence

Immunohistochemistry was performed using a rabbit anti-α smooth muscle actin (α-SMA, Boster, Wuhan, China) at a dilution of 1:150 or anti-PCNA antibody (Boster) at a dilution of 1:200. Briefly, the sections were de-paraffinized, rehydrated, and antigen retrieved and then treated with 0.3% hydrogen peroxide to block the endogenous peroxidase activity. Next, the sections were washed with tap water and PBS and then incubated in 3% bovine serum albumin (BSA) at the room temperature for 1 h and with primary antibodies at 4°C overnight. After that, the sections were washed with PBS three times and incubated with a biotinylated secondary antibody (Vector Laboratories, Burlingame, CA, United States) at room temperature for 1 h and subsequently incubated with ABC solution (Vector Laboratories) for 30 min at room temperature in the dark. Color development was assessed using the diaminobenzidine substrate, and the sections were finally counterstained with hematoxylin and reviewed under a light microscope. Muscularization of the vessel wall was quantified by percent α-SMA staining in the vessel, which was defined as follows: non-muscularized: < 5%, partially muscularized: 5–50%, and fully muscularized: > 50% (Dahal et al., 2010).

To assess the size of the myocardial cells, frozen sections, 4 μm in thickness, were used to assess superoxide generation by fluorescence immunolabeling using wheat germ agglutinin (Alexa Fluor^®^488 Conjugate, Invitrogen, Carlsbad, CA, United States). The nuclei were stained with 4′,6-diamidino-2-phenylindole (DAPI; Sigma-Aldrich, ST Louis, MO, United States). Fifteen sections from each animal were evaluated.

### Cell Culture and Treatment

A rat lung macrophage line NR8383 was obtained from American Type Culture Collection (Manassas, VA, United States) and cultured in the F12K culture medium (Invitrogen, Carlsbad, CA, United States) supplemented with 15% fetal bovine serum (FBS, Gibco, Australia), 2 mmol L-glutamine, L-100 U/ml penicillin, and 100 μg/ml streptomycin in a humidified incubator with 95% air and 5% CO_2_ at 37°C atmosphere. For cell treatment, the cells were grown for 3 days and then incubated with the growth medium containing 10 μM of PNU-282987 for 24 h according to a previous study (Liu et al., 2017) in the presence or absence of primed ultra pure lipopolysaccharide (LPS; 200 ng/ml) and adenosine triphosphate (ATP; 1 mmol/l) (Lu et al., 2014). After that, the cell supernatant and lysates were separately harvested and analyzed.

### Enzyme-Linked Immunosorbent Assay (ELISA)

Levels of tumor necrosis factor alpha (TNF-α), as well as interleukin (IL)-1β, IL-6, and IL-18 of lung tissues were assayed using their ELISA kits (R&D Systems, Minneapolis, MN, United States) according to the manufacturer’s instructions. In brief, rat lung tissues were homogenized in a lysis buffer, and after centrifugation, the supernatants were collected and subjected to ELISA, while the cell culture-conditioned media were also collected for ELISA, respectively.

### Quantitative Reverse Transcriptase-Polymerase Chain Reaction (qRT-PCR)

Total RNA was isolated from 10 mg each of snap-frozen lung tissues using the TRIZOL Reagent^®^(Invitrogen) and quantified using a NanoDrop 2000 Spectrophotometer (Thermo Scientific, Rockford, IL, United States) and reversely transcribed into cDNA using a PrimeScript^®^RT reagent Kit (TaKaRa, Dalian, China) according to the manufacturers’ instructions. *q*PCR was performed using the SuperScript III Platinum SYBR Green Two-step *q*RT-PCR Kit (TaKaRa, Dalian, China) in the ABI PRISM^®^7500 qPCR System (Applied Biosystems, Foster City, CA, United States) according to the manufacturer’s protocol. The primers for α7nAChR (GenBank #NM_012832) were 5′-GCAAAGAGCCATACCCAG-3′ and 5′-CAGCAAGAATACCAGCAGAG-3′; GAPDH (GenBank #M17701), 5′-TCCTGCACCACCAACTGCTTAG-3′ and 5′-AGTGGCAGTGATGGCATGGACT-3′. The relative level of α7nAChR mRNA was calculated using the 2^-ΔΔCt^ method after normalization to GAPDH mRNA.

### Western Blot

Snap-frozen lung tissues (approximately 20 μg) and lung macrophages were homogenized in a lysis buffer containing 150 mM NaCl, 50 mM Tris-HCl, pH 7.4, 1 mM EDTA, 1% Triton X-100, 1% sodium deoxycholate, 0.1% SDS, and protease inhibitors on ice for 30 min and then centrifuged at 12,000 rpm at 4°C for 10 min to collect the supernatants. Equal amounts of protein samples were separated in 10% sodium dodecyl sulfate-polyacrylamide gel electrophoresis (SDS-PAGE) gels and transferred onto nitrocellulose membranes. For Western blotting, the membranes were first blocked in skimmed milk solution at room temperature for 1 h and then incubated at 4°C overnight with the following primary antibodies, i.e., anti-α7nAChR (1:200, sc-58607; Santa Cruz Biotechnology, Santa Cruz, CA, United States), anti-NLRP3, anti-caspase-1, anti-procaspase-1, and anti-IL-1β (all from Cell Signaling Technology, Danvers, MA, United States and diluted at 1:1000). All antibodies were diluted in 5% BSA in Tris-buffered saline-01% Tween 20 (TBST). On the next day, the membranes were washed with TBST three times and further incubated with horseradish peroxidase-conjugated secondary antibody (1:5000; ZSGB-Bio, Beijing, China) for 1 h at room temperature. The positive protein bands were developed by incubation with the enhanced chemiluminescence reagents and exposed to X-ray films using a gel imaging system (UVP, Upland, CA, United States). The expression of each protein was then quantified using Image J software, v1.5.0 (National Institutes of Health, Bethesda, MD, United States) by normalization to GAPDH levels.

### Statistical Analysis

All data are summarized as the mean ± standard error of the mean (SEM) of three independent experiments and statistically analyzed using SPSS 17.0 software (SPSS, Inc., Chicago, IL, United States). The data comparison between the two model groups was analyzed using an unpaired Student’s *t*-test (following a normal distribution), while data comparisons among these four groups was assessed using a one-way analysis of variance and followed by the Newman–Keuls multiple comparison test for normally distributed data. Kaplan–Meier curves were used to compare survival between different groups, and the log rant test was used to generate a *P*-value. All statistical analyses were two-tailed, and a significant difference was defined as *P* < 0.05.

## Results

### Downregulation of α7nAChR Expression and PNU-282987-Activating α7nAChR in MCT Rat Lung Tissues

**Figure 1:**
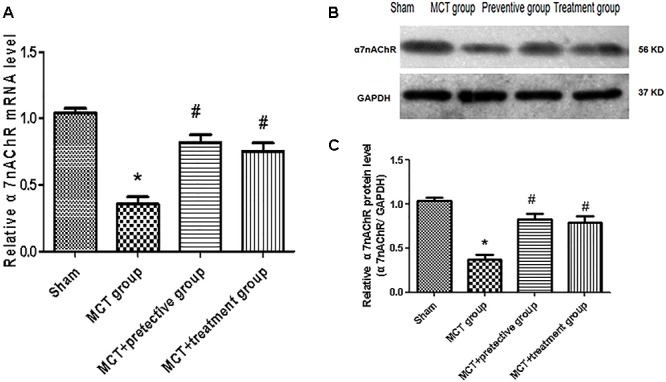
Effect of PNU-282987 treatment on α7nAChR mRNA and Protein expression in the lung specimen. **(A)** Fluorescence quantitative real-time polymerase chain reaction (RT-*q*PCR) showing that the relative level of α7nAChR mRNA was inhibited in the MCT group compared with the sham group, while α7nAChR mRNA expression in the PNU-282987 prevention and treatment rats were higher than those of the MCT group. **(B)** Representative western blot showing that the PNU-282987 upregulated MCT-induced PH α7nAChR (56 kDa) inhibition. **(C)** Relative protein levels of α7nAChR were determined after normalization to GAPDH among the four groups. ^∗^*P* < 0.05 versus the sham group and ^#^*P* < 0.05 versus the MCT group. Results shown are from one experiment (sham group, *n* = 15; MCT group, *n* = 8; MCT+protective group, *n* = 13; MCT+treatment group, *n* = 11).

In this study, we established the MCT rat model to mimic human PH and then treated these rats with the selective α7nAChR agonist PNU-282987 to assess α7nAChR expression and activity in MCT-treated rat lung tissues. Our data showed remarkable down-regulation of α7nAChR mRNA and protein in the MCT-treated rat lung tissues compared with the sham group (*P* < 0.05; Figure 1). In contrast, PNU-282987 treatment significantly up-regulated α7nAChR mRNA and protein in both the treatment and prevention groups (*P* < 0.05; Figure 1).

### Effect of PNU-282987 on Survival of Rats With MCT-Induced PH

**Figure 2:**
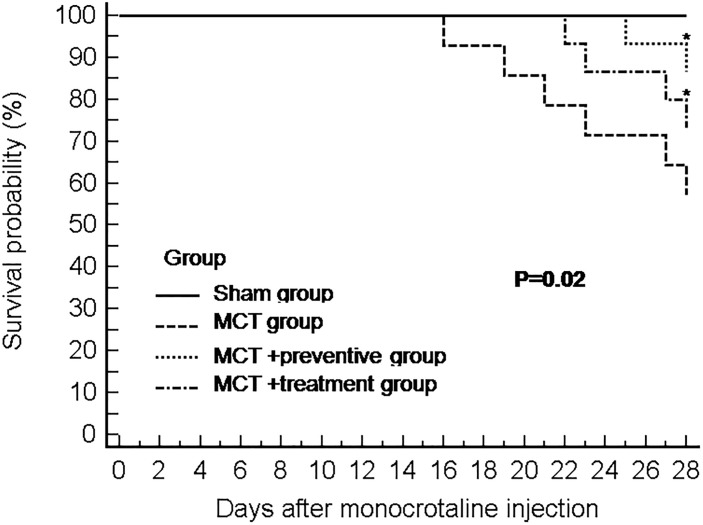
The Kaplan–Meier curves of MCT rats with or without PNU-282987 treatment. Kaplan–Meier survival curves demonstrate that significantly higher mortality in the MCT group than the sham group. Meanwhile, both the PNU-282987 prevention and treatment rats had significantly higher survival rates than those in the MCT group (*P* = 0.02).

Our animal experiments had 15 rats per group, and at the end of 28 days after MCT injection, 15, 8, 13, and 11 rats survived in the sham, MCT, MCT plus preventive, and MCT plus treatment groups, respectively. As shown in Figure 2, PNU-282987 treatment significantly increased the survival of rats in the MCT+α7nAChR preventive and treatment groups compared with that of the MCT vehicle-treated group (*P* < 0.05).

### Effect of PNU-282987 on Prevention and Treatment of PH-Induced Hemodynamic Changes in a MCT Rat Model

**Figure 3:**
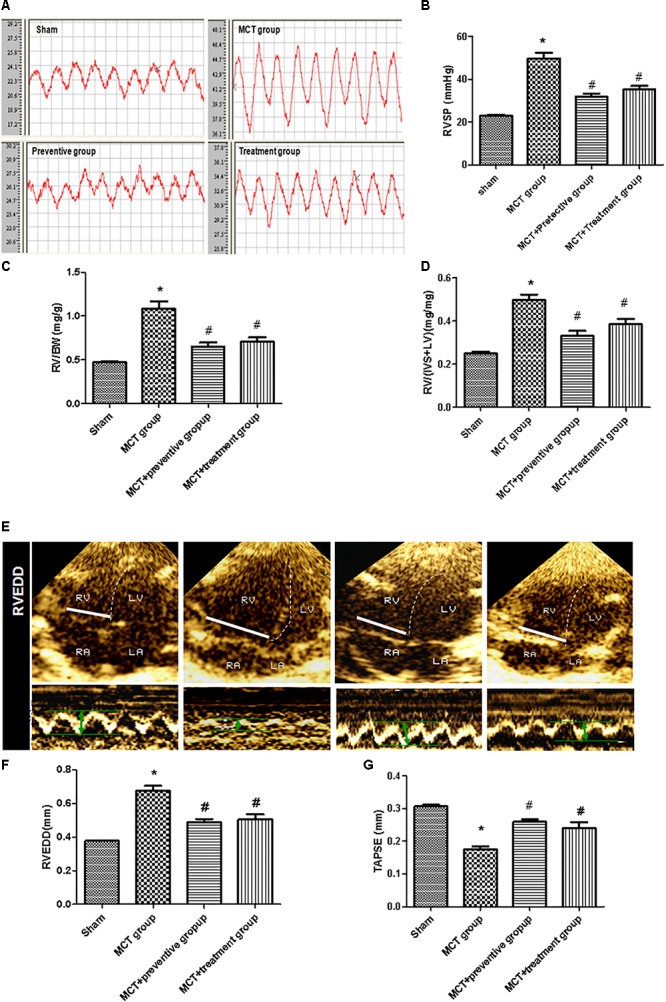
Inhibition of MCT-induced increase in right ventricular systolic pressure, hypertrophy, enlargement, and dysfunction by PNU-282987. **(A)** The representative right ventricular hemodynamic waves are presented in the sham, MCT, prevention, and treatment groups. **(B)** Right ventricular systolic pressure (RVSP) was determined. **(C,D)** RV hypertrophy was determined by RV weight normalized to body weight (BW) and the ratio of RV/[Left ventricle (LV) + septum (S)] among the four groups, respectively. **(E)** RV end diastolic dimension (RVEDD) and tricuspid annular plane systolic excursion (TAPSE) measured by echocardiography. **(F,G)** Comparison of the RVEDD and TAPSE by echocardiography in different groups. The data are summarized as means ± SD. ^∗^*P* < 0.05 versus the sham group and ^#^*P* < 0.05 versus the MCT group. Results shown are from one experiment (sham group, *n* = 15; MCT group, *n* = 8; MCT+protective group, *n* = 13; MCT+treatment group, *n* = 11).

We then assessed hemodynamics and histology data on these rats and found that the MCT-treated rats developed severe PH due to a significant elevation of the RV systolic pressure and thickened the vessel walls compared with those of the sham group (*P* < 0.05; Figure 3A,B), which is consistent with previous studies (Kim et al., 2017); however, PNU-282987 treatment effectively suppressed the MCT-induced increase in the RV systolic pressure (*P* < 0.05; Figure 3A,B). The RV structure and function are vital indirect markers for the major determinant of functional state and prognosis in PH (Ryan and Archer, 2014). Thus, we measured the RV/BW, RV/(LV + S), RVEDD and TAPSE and compared with the sham group. The RV/BW and RV/(LV + S) were significantly elevated in the MCT-treated rats (*P* < 0.05), indicating RV hypertrophy; however, such a MCT-induced RV hypertrophy was prevented by or treated with PNU-282987 (*P* < 0.05; Figure 3C,D). Our echocardiography data also showed enlarged RVEDD and decreased TAPSE in MCT-treated rats (*P* < 0.05), but this was significantly improved by PNU-282987 therapy (*P* < 0.05; Figure 3E–G). These data indicate that PNU-282987 is useful in preventing and treating PH.

### Effect of PNU-282987 on Prevention and Treatment of Pulmonary Vessel Remodeling in a MCT Rat Model

**Figure 4:**
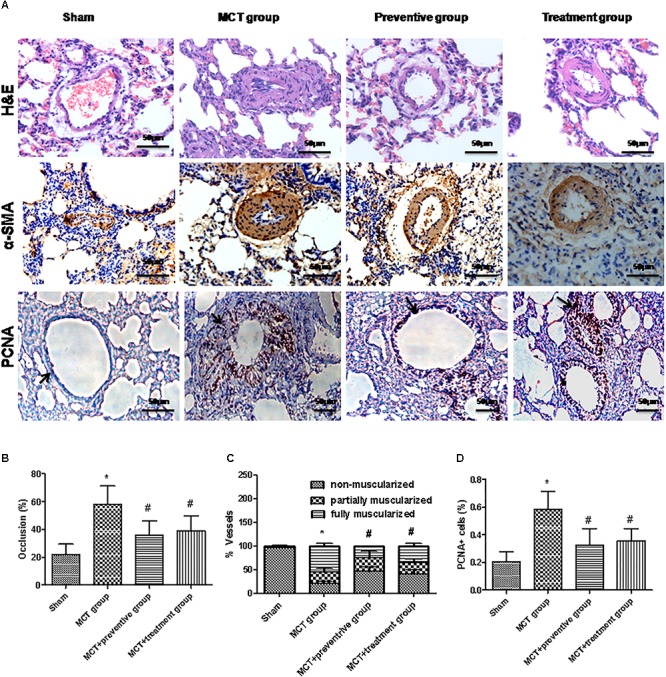
PNU-282987 treatment prevents and reveres pulmonary vascular remodeling. **(A)** Representative images of hematoxylin and eosin (HE), immunostaining of alpha-smooth muscle actin (α-SMA) and proliferating cell nuclear antigen (PCNA) from the lung in the sham, MCT, prevention and treatment groups. HE shows the thickness of the pulmonary artery. Brown staining with α-SMA indicates pulmonary artery smooth muscle, whereas brown-stained cells with PCNA represent proliferating pulmonary artery smooth muscle. The arrow indicates PCNA positive cells of the pulmonary artery. Occlusion (%), %vessels and PCNA positive cell (%) were calculated in a millimeter from 10 separate images of different fields. Original magnification: x400. **(B)** Graph showing the percentage of the median thickness of the arteriole. **(C)** Graph showing percentage of muscularization after α-SMA immunostaining. **(D)** Graph showing the percentage of PCNA positive cells. The data are summarized as means ± SD. ^∗^*P* < 0.05 versus the sham group and ^#^*P* < 0.05 versus the MCT group. Results shown are from one experiment (sham group, *n* = 15; MCT group, *n* = 8; MCT+protective group, *n* = 13; MCT+treatment group, *n* = 11).

Pulmonary arterial hypertension is associated with vascular remodeling, including muscularization and the occlusion of the pulmonary arteries (Huang Z. et al., 2016). In this study, we first confirmed these changes in MCT rats and assessed the effect of PNU-282987 prevention and treatment on pulmonary vessel remodeling in these MCT rats. Our data showed that MCT induced an increase in the muscularization and occlusion of the pulmonary arteries in MCT rats compared with the sham rats, whereas PNU-282987 treatment significantly reduced the occlusion rates and muscularization of the pulmonary arteries compared with the control rats (*P* < 0.05; Figure 4A–C). Immunohistochemically, the expression of PCNA, a proliferation marker, was significantly upregulated in pulmonary arterial smooth muscle cells in the walls of small pulmonary arteries in the MCT-treated vehicle group compared to the sham rats (*P* < 0.05; Figure 4A,D), whereas PNU-282987 treatment significantly reduced the number of PCNA-positive cells (*P* < 0.05; Figure 4A,D).

**Figure 5:**
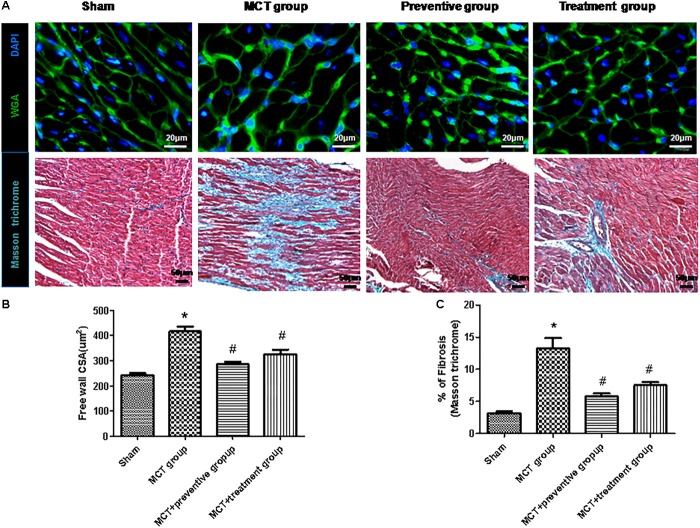
PNU-282987 on regulating cardiomyocyte size and collagen deposition in the right ventricle of MCT rats. **(A)** Representative images of the wheat germ agglutinin and Masson trichome staining of the right ventricle (RV), used to visualize and quantify the cardiomyocyte cross-sectional area and myocardial interstitial fibrosis in RVs, respectively, in the sham, MCT, MCT + PNU-282987 prevention and treatment groups. Myocytes are labeled with wheat germ agglutinin (green), nuclei are stained with DAPI (blue), original magnification: x400. Fibrosis presented as the blue stained area in the myocardium. Original magnification: x200. Images were observed in one millimeter from 10 separate images of different fields. **(B)** Quantification analysis of the cardiomyocte cross-sectional area (μm^2^). **(C)** Quantification analysis of interstitial fibrosis (%). The data are summarized as means ± SEM. ^∗^*P* < 0.05 versus the control group and ^#^*P* < 0.05 versus the MCT group. Results shown are from one experiment (sham group, *n* = 15; MCT group, *n* = 8; MCT+protective group, *n* = 13; MCT+treatment group, *n* = 11).

Furthermore, myocyte hypertrophy and tissue fibrosis are features of RV remodeling in PH (Kim et al., 2017). In this study, we found that the myocyte cross-sectional areas and interstitial collagen deposition were significantly increased in the MCT-treated vehicle group compared to the sham animals (*P* < 0.05; Figure 5), whereas PNU-282987 treatment showed reduced myocyte size and interstitial collagen deposition compared with the sham group (*P* < 0.05; Figure 5).

### Effect of PNU-282987 Prevention and Treatment on the Expression of NLRP3 Inflammasome Markers in a MCT Rat Model

**Figure 6:**
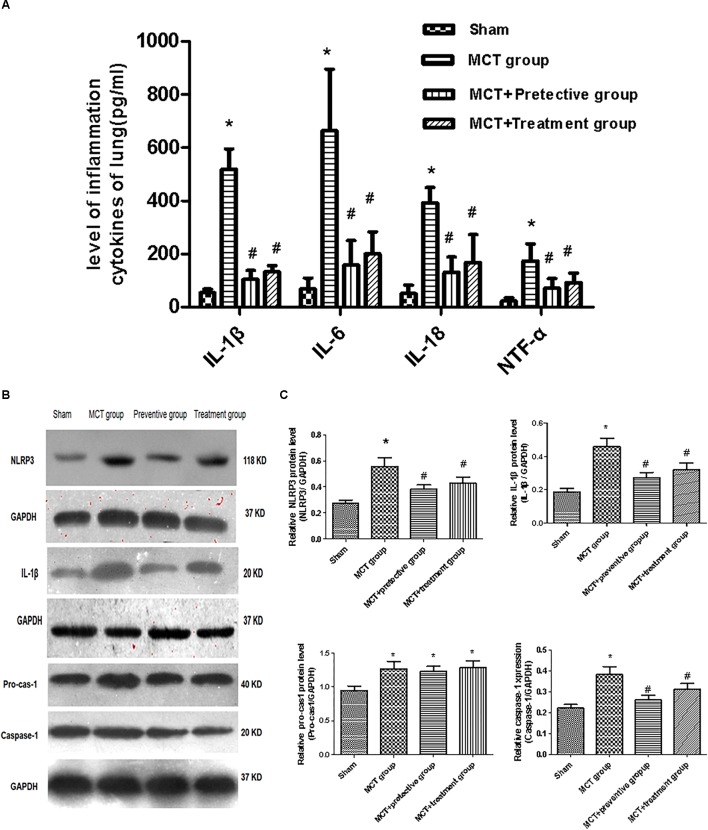
PNU-282987 therapy attenuated inflammation and blunted activation of NLRP3 inflammasome in lung tissues of MCT-induced PH rats. **(A)** Inflammation cytokines levels of IL-1β, IL-6, IL-18, and TNF-α were measured via ELISA in the sham, MCT, MCT + PNU-282987 prevention and treatment groups. **(B)** Representative western blot showing the PNU-282987 down-regulated lung MCT-induced PH induced activation of NLRP3 (118 kDa), IL-1β (20 kDa), Pro-casp-1 (40 kDa), and Caspase-1 (20 kDa) in lung tissue. **(C)** Quantification of western blot of NLRP3, IL-1β, Pro-casp-1, Caspase-1 in the four groups of rats, respectively. The data are expressed as means ± SD. ^∗^*P* < 0.05 versus the sham group and ^#^*P* < 0.05 versus the MCT group. Results shown are from one experiment (sham group, *n* = 15; MCT group, *n* = 8; MCT+protective group, *n* = 13; MCT+treatment group, *n* = 11).

We then assessed the levels of inflammatory cytokines, including IL-1β, IL-6, IL-18 and TNF-α, which have been shown to participate in pulmonary vascular remodeling (Rabinovitch et al., 2014; Gurtu and Michelakis, 2015). As demonstrated in Figure 6A, the levels of these cytokines were increased in the lung tissues of MCT rats, whereas PNU-282987 treatment significantly down-regulated their levels.

Furthermore, we also explored whether the NLRP3 inflammasome and the potential downstream signaling are involved in regulating inflammation cytokines by detecting the NLRP3 inflammasome markers, including NLRP3, pro-cas-1, caspase-1, and IL-1β. We found that the expression of NLRP3, caspase-1, and IL-1β was significantly upregulated in MCT rats compared with sham rats (*P* < 0.05; Figure 6B,C), whereas their upregulation was blunted by PNU-282987 prevention and treatment (*P* < 0.05; Figure 6B,C). However, no change in pro-cas-1 expression between the MCT, MCT + PNU-282987 preventive and treatment groups (*P* > 0.05; Figure 6B,C).

### Effect of PNU-282987 on the Reduced Expression and Secretion of Inflammation Cytokines and the Activation of the NLRP3 Inflammasome in Rat Lung Alveolar Macrophages

**Figure 7:**
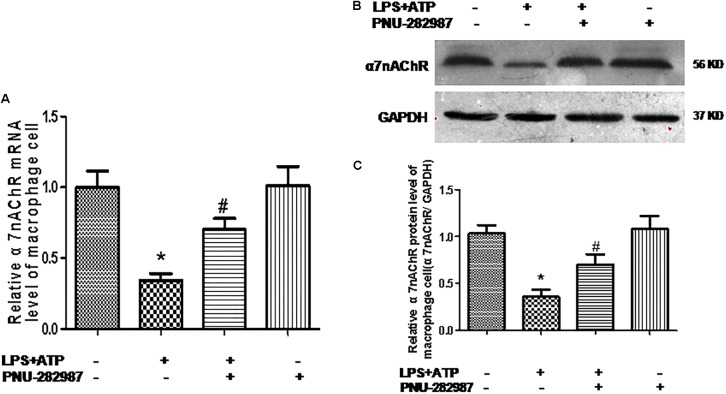
PNU-282987 treatment activation of α7nAChR mRNA and protein in lung macrophages. **(A)** Fluorescence quantitative real-time polymerase chain reaction (RT-PCR) showing the relative level of α7nAChR mRNA in lung macrophages treated with or without LPS+ATP, and PNU-282987. **(B)** Representative western blot showing the expression of α7nAChR (56 kDa) protein in lung macrophages treated with or without LPS+ATP, and PNU-282987. **(C)** Relative protein levels of α7nAChR were determined after normalization to GAPDH. LPS, lipopolysaccharide; ATP, adenosine-triphosphate. Data were accumulated from three independent assays. The data are expressed as means ± SD. ^∗^*P* < 0.05 versus the control cells and #*P* < 0.05 versus LPS+ATP cells.

**Figure 8:**
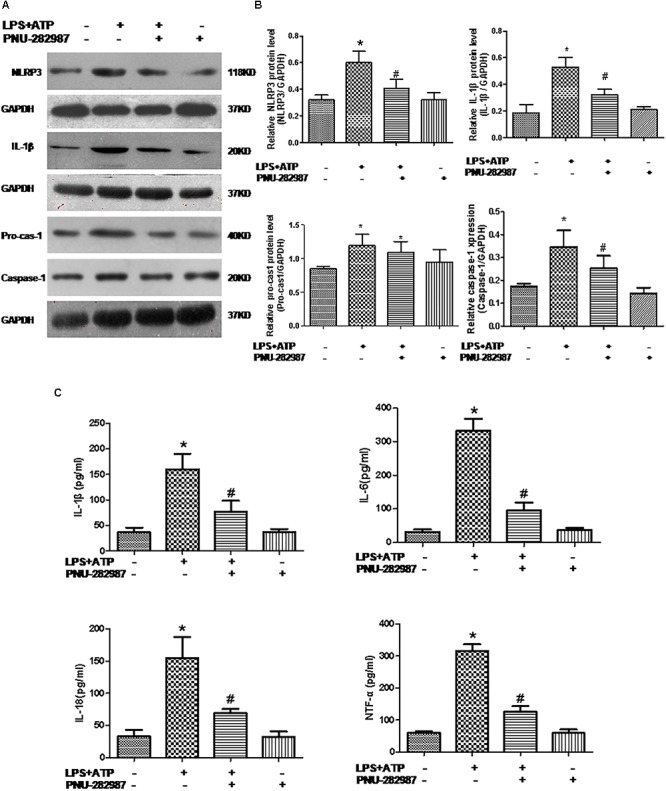
PNU-282987 treatment suppressed inflammation and downregulated NLRP3 inflammasome in LPS+ATP-treated lung macrophages. **(A)** Representative western blot showing NLRP3 (118 kDa), IL-1β (20 kDa), Pro-casp-1 (40 kDa), and Caspase-1 (20 kDa) protein expression in lung alveolar macrophages treated with or without LPS+ATP, and PNU-282987. **(B)** Relative protein levels of NLRP3, IL-1β, Pro-casp-1, and Caspase-1 were determined and normalized to GAPDH. **(C)** Secretion of the inflammatory cytokines (IL-1β, IL-6, IL-18, and TNF-α) in the four groups of lung macrophages were determined via ELISA. Data were accumulated from three independent assays. LPS, lipopolysaccharide; ATP, adenosine-triphosphate. The data are summarized as means ± SD. ^∗^*P* < 0.05 versus the control group and^#^*P* < 0.05 versus the LPS+ATP group.

Macrophages are one of the most abundant cell types in the lungs and proposed as a key pathogenic driver of pulmonary inflammation and PH (Florentin et al., 2018; Willis et al., 2018). Previous studies revealed that macrophage functions were regulated by the cholinergic anti-inflammatory pathway in neurons, the cardiovascular system, and infectious lung diseases (Yu et al., 2013; Ke et al., 2017; Liu et al., 2017; Wang J. et al., 2018). In this study, we further verified the participation of macrophages in PH development and found that LPS+ATP induced significant decreases in the expression of α7nAChR but induced the expression of NLRP3 inflammasome markers (NLRP3, caspase-1, and IL-1β) in a lung macrophage cell line (*P* < 0.05; Figure 7, 8A,B), PNU-282987 treatment markedly upregulated the expression of α7nAChR but suppressed the expression of NLRP3 inflammasome markers in macrophages (*P* < 0.05; Figure 7, 8A,B), whereas there was no significant difference between the control + PNU-282987 and control cells (*P* < 0.05; Figure 7, 8A,B).

Furthermore, our ELISA data revealed the significant induction of inflammatory cytokines (IL-1β, IL-6, IL-18, and TNF-α) in the LPS+ATP cells compared with the control cells (*P* < 0.05; Figure 8C), whereas their levels downregulated in the LPS+ATP+PNU-282987 cells, although there was no significant difference between the control+ PNU-282987 and control cells (*P* < 0.05; Figure 8C).

## Discussion

In this study, we obtained promising results for α7nAChR agonist, PNU-282987, therapy in MCT-induced PH in rats in prevention and treatment settings. We found that (1) The expression of α7nAChR was reduced in the lung tissues of MCT rats; (2) PNU-282987 treatment induced α7nAChR activation and attenuated and retarded MCT-induced lung vascular remodeling and PH, leading to RV protection and better animal survival; (3) PNU-282987 treatment downregulated the MCT-induced expression of the inflammatory markers (TNF-α, IL-6, IL-1β, and IL-18) in rat lung tissues. *In vitro* assay further reproduced these findings in rat lung macrophages. These effects appear to be associated with the modulation of the NLRP3 inflammasome.

The effect of PNU-282987 treatment on PH was first assessed by examining the change of hemodynamic values of the RV. Progressive increases in pulmonary vascular resistance pressure lead to RV hypertrophy, enlargement and dysfunction, and ultimately death (Tabima et al., 2012). In the present investigation, the calculated weight ratio of the RV/(LV+S) and RV/BW significantly decreased after PNU-282987 treatment, which was concurrent with a lower RV systolic pressure. Furthermore, PNU-282987 improved the survival rate both in the prevention and treatment groups. This was supported by preserved RV function, the inhibition of hypertrophy of cardiac myocytes and the interstitial fibrosis of RV myocardium, which have been considered prognostic markers in PH (Potus et al., 2015; Mendes-Ferreira et al., 2016).

Accumulated evidence indicates that inflammation plays a key role in the pathogenesis of PH in animal models and clinical patients (Rabinovitch et al., 2014; Gurtu and Michelakis, 2015). The histopathology for the pulmonary arterial system in PH has shown an impressive infiltration of inflammatory cells, especially macrophages, around remodeled vessels (Gurtu and Michelakis, 2015; Voelkel et al., 2016; D’Alessandro et al., 2018). Classically activated M1 macrophages elevated release of several inflammatory cytokines (IL-1β, TNF-α, IL-6, IL-18), thereby leading to vascular remodeling via matrix remodeling, as well as pulmonary vascular cell proliferation and migration in PH (Rabinovitch et al., 2014; Huang W. C. et al., 2016). The treatment of underlying inflammation has been shown to alleviate PH via inhibiting growth factor signaling, leading to the attenuation of the proliferative effects on pulmonary vascular cells. Consistent with the previous report (Zhao et al., 2017), we found that these inflammatory factors are obviously increased in an MCT-induced model.

Growing evidence has suggested a decisive role for inflammation responses, and reactions are also regulated by the sympathetic nervous system (SNS) (Kox et al., 2014; Lu et al., 2014). Previous studies demonstrated that the PNU-282987 induced suppression of SNS activity inhibited the macrophage release of pro-inflammation cytokines (Lu et al., 2014). α7nAChR agonists have been shown to be beneficial by attenuating inflammation in a murine model of radiation-induced and ischemia reperfusion-induced lung injury (Li et al., 2018; Mei et al., 2018). Similarly, our current study revealed that levels of α7nAChR mRNA and protein were downregulated in lung tissues of the MCT rats, indicating that α7nAChR activity could be important for controlling inflammation and pulmonary vascular remodeling. Thereafter, we further explored the role of α7nAChR in pulmonary vessel remodeling using the selective α7nAChR agonist PNU-282987. PNU-282987 has been broadly used to treat preclinical disease models, including myocardial injury (Hou et al., 2018), lung injury (Pinheiro et al., 2017), and nerve diseases (Vicens et al., 2017; Liu Q. et al., 2018). These studies indicated that the activation of the cholinergic anti-inflammatory pathway inhibited cytokine production and secretion for essential inflammation regulators via α7nAChR. The present study further validated the suppressive effect of PNU-282987 treatment on inflammation in MCT-induced PH via the activation of α7nAChR. Considering the key role of macrophages involved in the immunoregulation of PH, these results were further confirmed *in vitro* using PNU-282987 therapy to treat LPS+ATP-treated lung macrophages. We also provided direct evidence that PNU-282987 downregulated the LPS+ATP-induced inflammatory response (decreased levels of IL-1β, IL-6, TNF-α, and IL-18).

However, the precise mechanism by which PNU-282987 prevents PH and reduces inflammation factors remains to be determined. The NLRP3 inflammasome was identified as an important signaling pathway for inflammation and is modulated by the classical activation of macrophages (Liu F.Y. et al., 2018; Wang H. et al., 2018). Previous reports revealed NLRP3 inflammasome as a key factor in the signaling pathway mediating the inflammatory and remodeling effects in the context of PH (Tang et al., 2015; Yin et al., 2017). The activation of the NLRP3 inflammasome occurred after cell stress through the Toll-like receptor 4-ligand interaction and high extracellular ATP levels (Gaikwad et al., 2017). The NLRP3 inflammasome in alveolar macrophages could be activated after sensing lung alveolar stretching (Wu et al., 2013; van Lieshout et al., 2014). Our current study is in accordance with previous studies indicating the activation of the NLRP3 inflammasome in MCT induced PH. A recent study has reported that activating α7nAChR could inhibit the NLRP3 inflammasome, contributing to the control of neuroinflammation of auto-immune encephalomyelitis mice (Ke et al., 2017). Similarly, our study showed that the NLRP3 inflammasome was lower in rats receiving PNU-282987 therapy than rats treated with MCT alone. Moreover, LPS+ATP stimulation reduced the expression of α7nAChR but upregulated the NLRP3 inflammasome in lung macrophages, whereas PNU-282987 treatment upregulated α7nAChR expression but suppressed the NLRP3 inflammasome. These findings are in accordance with previous findings that α7nAChR activation suppressed the inflammatory response via NLRP3 inflammasome inhibition in BV2 microglia and peritoneal mouse macrophages (Lu et al., 2014; Ke et al., 2017).

The poor prognosis of patients afflicted by PH despite treatment with currently available vasodilator drugs makes the development of new treatment strategies imperative. Based on our current data, we speculate that the selective α7nAChR agonist, PNU-282987, could be a novel and promising approach to control and enhance the survival of patients with PH in a clinical setting. Recent studies of α7nAChR have proven to be effective in controlling connective tissue diseases (Stegemann et al., 2013; Fairley and Mathis, 2017). Thus, combined treatment consisting of prostacyclin, anti-remodeling agents (e.g., endothelin inhibitors) and anti-inflammation agents (like PNU-282987) might improve clinical prognosis for PH patients, especially those with connective tissue disease.

Though the α7nAChR agonist therapy provided valuable results in an MCT-induced PH rat model, concerns regarding the use of PNU-282987 in a clinical setting should be considered. First, our observations were performed in an MCT-induced rat model. Other PH models, such as hypoxia or high pulmonary blood flow model, must be further investigated. More importantly, there is a gap between the actual pathophysiology of rats and humans, and whether similar results can translate to humans must be confirmed. Second, our study is based on an inflammation theory by which the inhibition of inflammation flux could attenuate the PASMC proliferation. The direct effect of PNU-282987 on other cell types, e.g., smooth muscle cells and endothelial cells, cannot be completely ruled out. Third, the protocols developed here were designed to ameliorate MCT-induced PH in rats; thus, only one dose was used according to previous study (Ge et al., 2017). A dose-independent study requires further investigation.

## Conclusion

The current study demonstrates the potential for the α7nAChR agonist, PNU-282987, with respect to PH. PNU-282987 significantly prevents pulmonary artery remodeling and reduced RV systolic pressure, thereby inhibiting RV remodeling and dysfunction. These plausible effects of PNU-282987 in attenuating PH may be due to blunting inflammatory responses, which occur at least in part by downregulating the NLRP3 inflammasome pathway in macrophages. This research suggests that PNU-282987 may hold promise for future therapeutic applications in PH.

## Author Contributions

YD conceived, designed, performed the statistical analysis, and interpretation of data as well as prepared the manuscript. S-LG, BW, X-CG, Y-CZ, and J-QL carried out data collection. All authors have read and approved the final manuscript.

## Conflict of Interest Statement

The authors declare that the research was conducted in the absence of any commercial or financial relationships that could be construed as a potential conflict of interest.
